# Oxygen Persufflation in Liver Transplantation Results of a Randomized Controlled Trial

**DOI:** 10.3390/bioengineering6020035

**Published:** 2019-04-27

**Authors:** Anja Gallinat, Dieter Paul Hoyer, Georgios Sotiropoulos, Jürgen Treckmann, Tamas Benkoe, Jennifer Belker, Fuat Saner, Andreas Paul, Thomas Minor

**Affiliations:** 1 General, Visceral and Transplantation Surgery, University Hospital Essen, University Essen-Duisburg, 45147 Essen, Germany; anja.gallinat@uk-essen.de (A.G.); dieter.hoyer@uk-essen.de (D.P.H.); georgios.sotiropoulos@uk-essen.de (G.S.); Juergen-Walter.treckmann@uk-essen.de (J.T.); tamas.benkoe@uk-essen.de (T.B.); jennifer.belker@uk-essen.de (J.B.); fuat.saner@uk-essen.de (F.S.); andreas.paul@uk-essen.de (A.P.); 2 Surgical Research Department, General, Visceral and Transplantation Surgery, University Hospital Essen, University Essen-Duisburg, 45147 Essen, Germany

**Keywords:** liver transplantation, oxygen persufflation, reconditioning, randomized controlled trail

## Abstract

Oxygen persufflation has shown experimentally to favorably influence hepatic energy dependent pathways and to improve survival after transplantation. The present trial evaluated oxygen persufflation as adjunct in clinical liver preservation. A total of *n* = 116 adult patients (age: 54 (23–68) years, M/F: 70/46), were enrolled in this prospective randomized study. Grafts were randomized to either oxygen persufflation for ≥2 h (O2) or mere cold storage (control). Only liver grafts from donors ≥55 years and/or marginal grafts after multiple rejections by other centers were included. Primary endpoint was peak-aspartate aminotransferase (AST) level until post-operative day 3. Standard parameters including graft- and patient survival were analyzed by uni- and multivariate analysis. Both study groups were comparable except for a longer ICU stay (4 versus 3 days) of the donors and a higher recipient age (57 versus 52 years) in the O2-group. Serum levels of TNF alpha were significantly reduced after oxygen persufflation (*p* < 0.05). Median peak-AST values did not differ between the groups (O2: 580 U/l, control: 699 U/l). Five year graft- and patient survival was similar. Subgroup analysis demonstrated a positive effect of oxygen persufflation concerning the development of early allograft dysfunction (EAD), in donors with a history of cardiopulmonary resuscitation and elevated ALT values, and concerning older or macrosteatotic livers. This study favors pre-implantation O2-persufflation in concrete subcategories of less than optimal liver grafts, for which oxygen persufflation can be considered a safe, cheap and easy applicable reconditioning method.

## 1. Introduction

The worldwide growing organ shortage led to an adjustment of thresholds to accept organs for transplantation. In the field of liver transplantation the numbers of donors aging > 65 years has increased more than ten-fold from 1991 to 2001 in the United Network for Organ sharing as well as the European Liver Transplant registry [1]. Likewise acceptance of steatotic organs and other risk afflicted organs has been increasing. Former studies demonstrated inferior outcomes for such organs [2,3,4]. Organs derived from suboptimal donors carry higher susceptibility of preservation/ischemia and reperfusion injuries, which ultimately results in higher rates of early allograft dysfunction after liver transplantation, a complication associated with reduced graft and patient survival [5,6]. 

Therefore, organ preservation techniques need to be adapted, contributing to the demands of suboptimal grafts. Optimized preservation techniques present a valuable opportunity to decrease ischemic organ injury and increase the number of viable donor organs and advance the total pool of organ donors. 

The attenuation of ischemic organ damage can be elegantly achieved by the provision of gaseous oxygen during static cold storage. Development of preservation damage most likely depends on adequate redox and intracellular signal homeostasis. Venous oxygen persufflation [7] is thought to replenish depleted cellular energy stores in a simple and applicable way. As the majority of preservation/reperfusion injury arises at the time of the warm reperfusion of the organ [8,9], an end-ischemic adaption of the preservation method carries the possibility to prime the organs for this critical period of the transplantation process. Indeed, the optimal treatment time for a hypothermic reconditioning of cold stored liver grafts by gaseous oxygen persufflation was previously evaluated in a large animal model, demonstrating the best results after 2 h of end-ischemic reconditioning [10,11]. The mechanism includes the stabilization of cell and organ integrity by reduction of ischemia induced failure of cellular autophagy [12], which leads to an increased regenerative potential of the cells during the reperfusion period to clear impaired cell organelles and reprocess denaturated proteins [13,14]. 

First clinical applications of this end-ischemic organ persufflation demonstrated feasibility and safety in the clinical setting [15]. Subsequently, Khorsandi and coworkers [16] were able to confirm gaseous oxygen insufflation to improve hepatic energy homeostasis at the end of ischemic preservation in human discard livers. 

Based on these encouraging observations, a randomized controlled trial was created to systematically address, whether 2 h of gaseous oxygen persufflation of the isolated liver graft immediately prior to transplantation will improve early graft function upon reperfusion and mitigate adverse effects associated with preservation/reperfusion injury as compared to standard cold storage. 

## 2. Patients and Methods

### 2.1. Study Design

The present study was carried out at a single center. It was designed as randomized, controlled, single blinded clinical study and comprised two arms (treatment versus control) (ISRCTN00167887). The study was approved by the local ethics committee (Ethics committee University Hospital Essen, AZ 09-4281) and followed the Declaration of Helsinki. The study was supported by the German Research Foundation (DFG MI 470/14/2).

### 2.2. Study Population

Only patients who met all inclusion and no exclusion criteria were included in the trial. 

Inclusion criteria for the allografts were met when the organ was allocated by the “rescue offer” mechanism by EUROTRANSPLANT (see below) or when donor age was 55 years or older. (In contrast to the originally foreseen donor age criterion of >65 years, the required donor age has been reduced from 65 years to 55 years in order to cope with unexpectedly low numbers of organ offers at the time of enrollment and to safeguard the timely completion of the study. A respective official amendment been approved by the local authorities.) 

For the inclusion criteria of the liver transplant recipients the following requirements had to be met:

- Adult patients (>18 years of age)
- Recipients undergoing the first liver transplantation
- Willingness and ability to attend regular follow up examinations
- Written informed consent

Exclusion criteria for the recipients included high urgency listing, participation in other clinical trials and positivity for HIV. 

### 2.3. Study Procedures

After acceptance of organ offers all livers were inspected at the local transplant center by an experienced transplant surgeon. Liver zero-biopsies were done by the acting implant surgeon, whenever the macroscopic appearance of the liver deemed questionable. Thus, in some cases, livers were transplanted without prior histology.

When found to be suitable for transplantation randomization was initiated after verification of all inclusion and exclusion criteria. Randomization was technically realized by a web-interface organized by the center for clinical trials Essen with 1:1 randomization ratio as per computer-generated randomization schedule. Variable block sizes were used with patient level stratification for Model for End-Stage Liver Disease (MELD) score (3 groups: <20, 20–30, and >30). 

After randomization to one of the study arms all procedures strictly followed the study protocol: For the treatment group, donor livers were subjected to 2 h of venous systemic oxygen persufflation (OPAL) as detailed previously [15,17]. Shortly, allografts were stored in ice-cold preservation solution during the procedure and backtable preparation was carried out as usual. Additionally, a catheter was inserted into the suprahepatic vena cava. An atraumatic clamp temporarily closed the infrahepatic vena cava. Filtered (membrane pore size of 5 µm) and humidified oxygen gas was then introduced via the catheter in the suprahepatic caval vein at a pressure limited to 18 mmHg to avoid barotraumata of the vasculature. An endosufflator (WISAP GmbH Sauerlach, Germany), which was technically modified for the use of oxygen instead of carbon dioxide, was utilized. When persufflation is started postsinusoidal venules become dilated due to the pressure applied to the hepatic venous system and gas bubbles rise up via the portal vain. Additionally, small pinpricks are set with a 27 gauge needle into dilated venules at the periphery of the liver lobes that also allow the oxygen to leave the microvasculature [17,18]. In contrast to the machine perfusion technologies, no liquid perfusion is involved in the oxygenation of the liver tissue, that takes place exclusively by gaseous diffusion. The persufflation method hence does not require the use of additional oxygenators or disposable perfusion kits. 

For the control group livers were kept simply cold stored until implantation after usual backtable procedures. 

After transplantation all patients were observed for seven days on a daily basis. Additional follow up visits were carried out on the day of discharge, 3, 6, and 12 months after transplantation. Further follow up included common visits at our outpatient clinic. 

### 2.4. Objectives and Endpoints

Primary endpoint was the peak value of serum aspartate aminotransferase (AST) during the first three days after liver transplantation. Secondary endpoints were graft and patient survival, rate of re-transplantation, early allograft function (EAD, see below), ICU stay, time of postoperative ventilation and dialysis as well as morbidity according to Dindo-Clavien Classification Grade ≥ 3. Moreover serum levels of TNF-alpha were determined to investigate the impact of hypothermic reconditioning on pro-inflammatory upregulation after reperfusion. Serum samples taken one hour after reperfusion were analyzed using commercial ELISA kits on a fluorescence micro plate reader (Tecan, Grailsheim, Germany) according to the instructions of the manufacturer (R&D Systems, Wiesbaden, Germany).

### 2.5. Surgical Procedure and Immunosuppression

All organ procurements were carried out by specialized local teams according to the standards of the local procurement organizations within the different EUROTRANSPLANT regions. Orthotopic liver transplantation was performed with vena cava replacement and end-to-end-anastomosis of portal vein, hepatic artery and bile duct. All patients were treated at the ICU after transplantation. The perioperative care was similar in both groups as well as the concept of immunosuppression. Intravenous corticosteroids (1000 mg methylprednisolone) were applied intraoperatively. Postoperatively, tacrolimus (adjusted in accordance to the trough level of the drug) in combination with corticosteroids and mycophenolate mofetil were utilized. 

### 2.6. Definition of Rescue Allocation

Livers refused by more than three different centers for allocated candidates with the highest MELD scores on the national waiting list were characterized as “organ rescue offers”. These grafts were then either offered to the nearest center with a suitable recipient or allocated to the first center to accept them (multiple-refusal/competitive rescue offer procedure). “Organ rescue offers” were also occasionally encountered in instances of donor instability, prolonged cold ischemic times, or unfavorable logistic reasons.

### 2.7. Definition of Early Allograft Dysfunction (EAD)

Early Allograft Dysfunction (EAD) was defined as: Bilirubin ≥ 10 mg/dL on postoperative day 7 and/or INR ≥ 1.6 on postoperative day 7 and/or AST or ALT > 2000 IU/L within the first 7 days [19]. Each case was classified as “EAD” or “no-EAD.” 

### 2.8. Clinical Factors for Outcome Analysis

The following donor factors: age, gender, BMI, cause of death (cerebrovascular accident, hypoxia, trauma, others), cold ischemic time, ICU length of stay, biopsy proven steatosis (macrovesicular and microvesicular), organ protection solution used during the procurement (HTK, UW), last laboratory values (AST, ALT, gGT, Bilirubin, Creatinine, Serum Sodium, INR) and the Donor Risk Index (DRI) [20]—for the calculation of the DRI “race” was always set to “Caucasian”. The following recipient factors were analyzed: age, gender, BMI, etiology of liver disease, laboratory Model for End stage Liver Disease (MELD) score before transplantation, time for surgical procedure, warm ischemic time, hospitality stay, and rejection within 3 month. Charlson Co-morbidity index was calculated according to Charlson et al. [21].

### 2.9. Monitoring, Data Safety Monitoring Board

All trial related procedures were monitored and controlled by the center for clinical trials Essen (ZKSE), according to ICH-GCP guidelines. Additionally, an independent data safety monitoring board (DSMB), consisting of a clinician, scientist and statistician closely followed the proper conduct of the trial and all severe adverse events (SAE). SAE were defined as life threatening or deadly events or events that entail permanent injuries or require prolongation of hospital stay. None of the occurring complications (the frequency of which did not differ between the groups) gave reason for intervention by the safety board. 

### 2.10. Sample Size Calculation and Statistical Analysis

The sample size calculation was performed to detect a relative effect of p = 0.66 (which is comparable to a mean difference of ~0.6 in units of standard deviations of a standard normal distribution) for the primary endpoint (maximum AST value during the first three days after transplantation) with a power of 0.8 when significance is set to a = 0.05 (two-sided). A drop-out rate of 10% was assumed, resulting in a sample size of 58 patients per groups (total 116 patients). 

Data were expressed as mean and standard error of the mean or median and range values, as appropriate. Categorical variables were analyzed by chi-squared test. Continuous variables were analyzed by the Student t test or the Mann–Whitney U test. Treatment groups and clinical parameters were linked to the development of EAD after transplantation by univariable and multivariable logistic regression analysis censored for treatment groups. Factors with a *p*-value < 0.1 in either group were introduced into the respective multivariable model. Patient survival was calculated using the Kaplan–Meier method and compared with the log-rank test. Univariable and multivariable Cox Proportional Hazard Analyses were carried out to delineate independent predictors of patient survival. Long-term patient survival was censored for patient death and treatment arm in order to investigate the impact of study procedures on the early outcome after transplantation and intervention. *p* < 0.05 was considered to be statistically significant. Statistical analyses were performed using JMP (version 10.0.0 SAS, SAS Institute Inc., Cary, NC, USA) and SPSS (IBM SPSS Statistics 24, IBM^®^, Armonk, NY, USA).

## 3. Results

### 3.1. Recruitment and Follow Up

Patients were enrolled and transplanted from 09/2011 to 12/2013. According to the study protocol 116 patients were recruited. Of these, 57 patients were randomized into the treatment group and 59 patients randomized into the control group. One year follow up was 100%. Graft and patient survival were analyzed for a total of 5 years after transplantation.

Only one patient was lost to follow up with a functioning allograft after more than 2 years after transplantation. 

**Figure 1:**
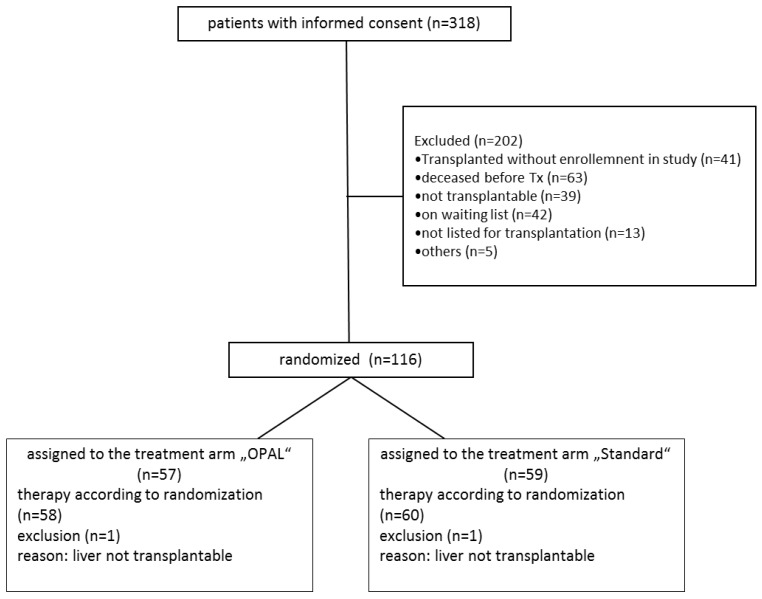
CONSORT diagram illustrating the study enrollment.

Median follow up time was 1466 days (1–2028 days). Enrollment of patients is shown in Figure 1. 

Following randomization 2 patients (1 in each treatment arm) were excluded as the donor organ has been considered inacceptable for transplantation after inspection by the responsible surgeon.

### 3.2. Donor, Recipient, and Perioperative Characteristics

Mean age of donor organs was 63 (±1.26) years. Half (50%) of the donors were male. Median donor ICU treatment before organ procurement was 3.0 (1–19) days. The median DRI was 1.8 ± 0.3. Cold preservation was applied for 452 ± 13.4 min. The warm ischemic time during the surgical procedure was 30 ± 0.6 min. 

Recipients had a mean age of 53.2 ± 0.8 years and were predominantly male (60.3%). Indications (or a combination of indications) for OLT included cirrhosis related to alcoholic cirrhosis (31.9%), viral hepatitis (26.7%), hepatocellular carcinoma (26.7%), NASH (6.9%) and others (25.9%). The mean labMELD before liver transplantation was 14.6 ± 0.6. Median duration of the surgical procedure was 257 (155–661) min.

Further details regarding donor, recipient and perioperative characteristics in both study arms are given in Table 1. 

In brief, clinically relevant characteristics were similar in both groups. Donor ICU stay was significantly shorter in the treatment group. Recipient age was significantly older in the treatment group. In both groups allocation was center based in 80%. Cold ischemia time was numerically longer in the treatment group (*p* > 0.05). 

### 3.3. Surgical Study Procedures

Oxygen persufflation was applied for 137 (103–205) min in the treatment group. Treatment with persufflation did not result in any serious adverse event or allograft loss. Minor bleeding was observed from the pinpricks. These were not clinically relevant and stopped spontaneously or after minimal electrocoagulation. Median duration of the surgical procedure was 260 (176–460) min in the treatment arm and 250 (155–661) min in the control group. Warm ischemia time was similar, being 30 (16–41) min in the persufflation group and 29 (20–65) min in the control group. Statistical differences regarding surgical study procedures were not observed between groups. 

Transfusion of packed red blood cells (PRBs) was low and the same in both groups: 16 patients (28%) in the treatment group were transfused with a median of 2 PRBs compared to 25 patients (42%) transfused with a median of 2 PRBs in the control group. 

### 3.4. Primary Endpoint

**Figure 2:**
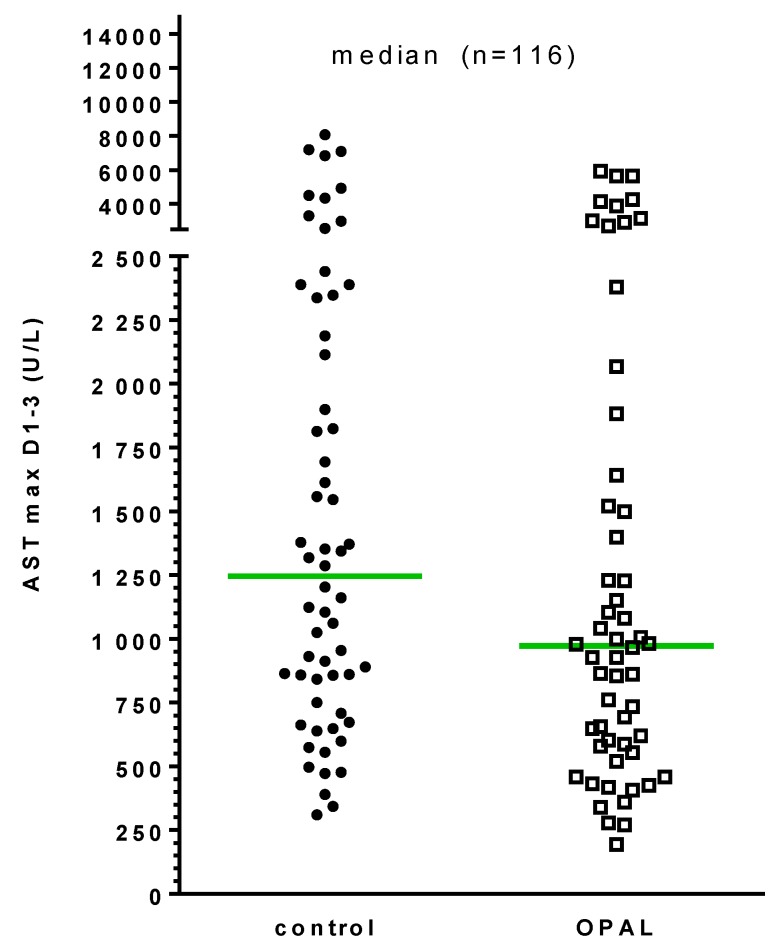
Peak values of AST during the first 3 days after transplantation.

Peak AST values within the first three days after liver transplantation were higher in the control group compared to the treatment group. However, this did not reach statistical significance (1246 (310–8064) versus 972 (194–17577), control versus OPAL; cf Figure 2).

### 3.5. Secondary Endpoints

For secondary endpoints several assessments of patient death and early graft function were compared between groups. Details are depicted in Table 2. 

Rates of 30-day mortality and In-hospital mortality were not statistically different between groups. Few retransplantations were necessary in the present study: One patient developed primary non-function and died after retransplantation. Another patient in the treatment group developed arterial thrombosis one month after transplantation and was successfully retransplanted. This patient is now well and alive. Statistical comparison of PNF and retransplantation rates was not performed due to the low number of events. Early Allograft Dysfunction occurred in every fifth to fourth patient in both groups. Rate of postoperative acute kidney failure and the necessity for hemodialysis was higher in the control group, but did not reach statistical significance. Postoperative complications ≥ Grade III according to Dindo-Clavien were similar in both groups. Surrogates of complicated clinical courses like length of ICU stay and length of hospital stay did not show differences among the groups. 

Serum levels of TNF alpha were found to be significantly reduced after oxygen persufflation:

11.1 ± 1.6 versus 5.9 ± 0.4 pg/ml; mean ± SEM, control versus OPAL *p* < 0.05.

### 3.6. Patient and Graft Outcome

Death censored graft survival in the treatment group was 89% after one, three, and five years. The control group demonstrated a graft survival of 87%, 84% and 82% after one, three, and five years, respectively (*p* > 0.05). 

Patient survival in the complete study cohort was 80% and 70% after one and five years, respectively. 

Patient survival in the treatment group was 77%, 74%, and 74% after one, three and five years. Accordingly, the control group demonstrated patient survival rates of 83%, 76%, and 66%. Comparison between groups demonstrated non-significant differences (*p* = 0.56). 

Overall cause of death in the recipients after transplantation was in descending order: Tumor recurrence (10.4%), sepsis (8.6%), and HCV reinfection (6.9%). 

### 3.7. Association of Clinical Parameters with Development of EAD

Logistic regression was performed to identify parameters associated with the development of EAD as a clinical marker for inferior allograft function. Results are displayed in Table 3. 

For the treatment group, no clinical parameter was delineated as independent predictor for development of EAD after liver transplantation. In contrast, history of cardiopulmonary resuscitation and last donor ALT were significantly and independently associated with the development of EAD in the control group. Summarizing, oxygen persufflation showed a positive effect concerning the development of EAD in the case of donors with history of cardiopulmonary resuscitation and of donors with elevated ALT levels.

### 3.8. Association of Clinical Parameters with Patient Survival

Cox proportional hazard analysis was performed to identify parameters associated with long term patient survival (Table 4). 

None of the analyzed factor demonstrated an independent association with the patient survival in the treatment group. On the contrary, two factors were significantly and independently associated with the patient survival in the control group: allograft macrosteatosis as a marker for graft quality and the MELD score of the recipients as a marker for severity of the underlying liver disease. 

Quite of interest in this analysis was the strong tendency of donor age with the patient survival in the control group. Further analysis demonstrated that an advanced donor age of more than 70 years was significantly associated with the patient survival in univariable analysis. This was not observed in the treatment group. Due to the retroactive character of this analysis, donor age > 70 years was not introduced in the multivariable cox proportional model.

These findings could be interpreted as a positive effect of the revitalization treatment in the case of older livers (from donors > 70 years of age) or macrosteatotic livers, proposing selection criteria for the use of this method in the corresponding instances.

### 3.9. Subgroup Analysis

**Figure 3:**
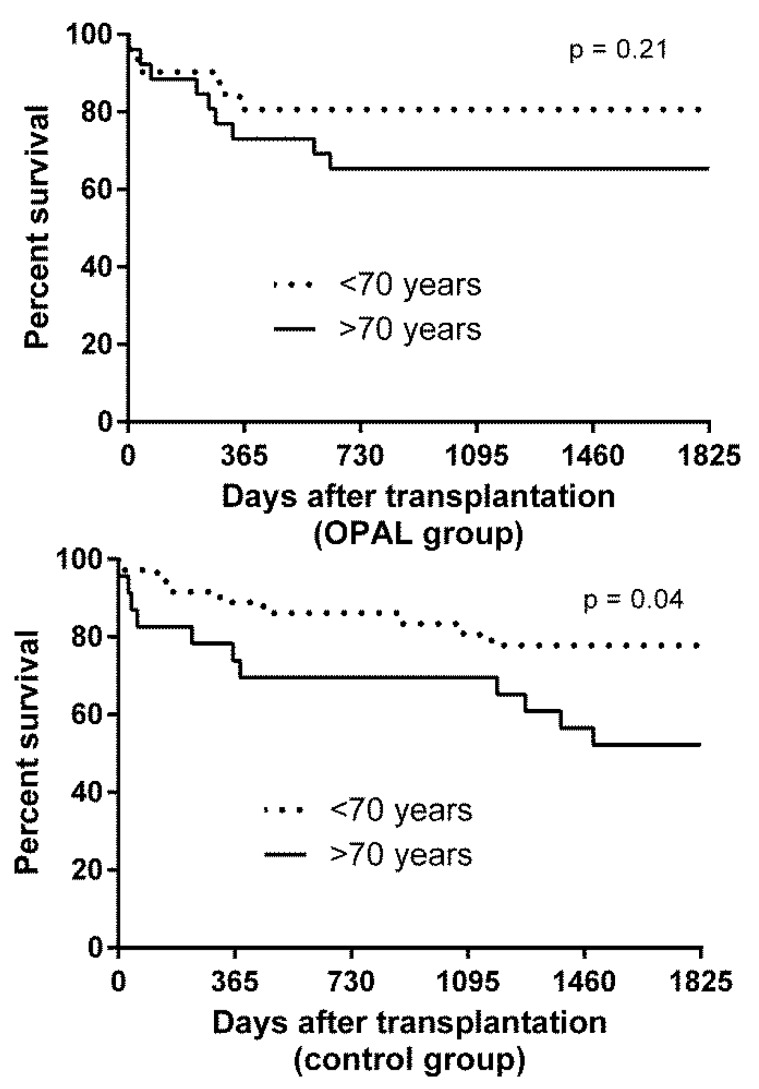
Five year patient survival in the treatment group (oxygen persufflation (OPAL)) and in the standard care group (control) according to recipient age (<70 years versus ≥70 years).

The impact of advanced donor age was further investigated in a subgroup analysis. Patient survival was compared in recipients transplanted with organs from donors with age > 70 years to recipients transplanted with organs from donors with age < 70 years in the treatment group (*n* = 26) and control group (*n* = 23), respectively. Results are depicted in Figure 3. 

In the treatment group patient survival was 80% after one and five years when transplantation was carried out with younger donors. In donors aged 70 or more the patient survival was 70% and 65% after one and five years, without statistical significant differences between groups. However, the same analysis in the control group demonstrated significantly worse survival after transplantation of allografts from donors aged 70 years or more with one and five year patient survival of 70% and 48%. Donors aged younger than 70 years led to a patient survival of 85% and 75% after one and five years, respectively. 

We also evaluated maximal serum values of AST in the population receiving macrosteatotic livers (>20%) and found an accentuated trend towards a benefit in the OPAL group: 987 (271–3016) U/l versus 2498 (890–4332) U/l. However, because of the small number of patients (*n* = 12 in the OPAL group, *n* = 6 in the control group) differences were not evaluated for significance. 

## 4. Discussion

This randomized controlled single center study investigated for the first time the impact of oxygen persufflation as adjunct in liver preservation on early allograft injury and dysfunction upon repefusion. The primary endpoint was aminotransferase peak of AST within the first three days after liver transplantation. While AST values were lower in the treatment group, statistical significance was not reached. 

Assessment of secondary endpoints, such as early allograft dysfunction, primary non-function, and patient survival, showed a positive effect of the treatment on the development of EAD in the case of donors with history of cardiopulmonary resuscitation and of elevated ALT levels. Benefits with regard to patient survival were also present for marginal liver grafts with macrosteatosis or originating from donors aged > 70 years.

Furthermore a moderate, but significant reduction of TNF-alpha release could be documented for the entire collective.

Thus, a distinct clinical benefit for application of retrograde oxygen persufflation as reconditioning tool immediately before liver transplantation could be delineated in concrete subcategories of particularly endangered donor grafts. This comes to confirm the corresponding literature from experimental studies, as the concept of retrograde oxygen persufflation in liver allograft reconditioning is based on scientifically high grade research which has been published in the past decades: Initial reports by Isselhard [22] and Ross [23] demonstrated convincing results in animal kidneys already in the 1970s. Subsequently, the first clinical pilot project was successfully initiated in renal transplantation [24]. Experimental applications in liver allografts followed thereafter [25,26]. This research demonstrated significant reduction of non-parenchymal cell injury and vascular endothelial dysfunction after cold preservation of the liver by gaseous oxygen [27] as well as reduction of proteolysis leading to improved functional outcome after transplantation [28]. Gaseous oxygenation resulted in normalization of vascular resistance and reduced release of hepatocellular enzymes. Further investigations showed prevention of functional and ultrastructural impairments by venous oxygen persufflation [29] in steatotic rat livers. 

In a porcine model, gaseous oxygen persufflation prevented primary non-function of livers after extended cold storage times and improved one week survival of the recipient from 0% up to 83% [11].

Based on these premises one might have expected a less equivocal result upon clinical usage oxygen persufflation. However, in contrast to the controlled experimental situation, a broader heterogeneity of included donor livers as well as recipient health status may influence the outcome after transplantation in the clinical setting.

Some of the donor organs might have had lesser needs for additional treatment than others, and, although distributed evenly across the groups, a notable fraction of not so marginal grafts might have obscured the benefit of oxygen persufflation in less resilient livers. 

Such effect would not have been predictable, as extensive human studies are needed to delineate the specific influence of preservation techniques on each group of allografts. The subgroup analysis presented in this studies might be taken as hint that oxygen persufflation might be most effective in older donor allografts. While the study was not powered to prove this, the influence of donor age on patient survival in the control group, which was not traceable in the treatment group, suggests reduced damage to the allograft after oxygen persufflation. 

In addition, results of multivariable studies demonstrated that history of donor cardiopulmonary resuscitation as well as elevated ALT levels in the donor contributed to development of EAD in the control group but not in the treatment group. This might indicate that after reconditioning of pre-damaged allografts, such risk factors lost their impact. The same was observed for patient survival: allograft macrosteatosis and MELD score were delineated as risk factors only in the control group. Against the background of similar patients and allografts transplanted in both groups, this resembles a surrogate of improved allografts after treatment by oxygen persufflation. 

It should be of interest that the data at hand demonstrates excellent safety of the method applied. Serious adverse events related to treatment were not observed and all endpoints were qualitative similar to the common standard of cold storage. 

Organ preservation is a science virtually lacking relevant clinical progress over more than 20 years. 

Nowadays, several approaches of machine perfusion have been taken from experimental projects to first clinical applications [30,31,32]. These studies demonstrated feasibility and safety of such new methods and suggested beneficial effects on clinical outcomes like peak of aminotransferase after transplantation, development of EAD and maybe even biliary complications. Most importantly, these pilot projects led to the initiation of randomized controlled trials comparing these new methods with static cold storage. So far, no clear benefits in term of graft and patient survival could be demonstrated. 

This again underscores the importance to identify more precisely those subgroups of allografts that need or may benefit from reconditioning measures and those who will not. 

Compared with the more sophisticated methods of machine perfusion, the simple insufflation of gaseous oxygen excels by its ease of use and the unmatched cost-effectiveness may furthermore allow for a less critical application in attempt to rescue questionable liver grafts.

## 5. Conclusions

In conclusion, this randomized controlled trial demonstrated safety of venous oxygen persufflation of liver allografts immediately before transplantation as reconditioning tool. A clinical benefit could be demonstrated in concrete subcategories of less than optimal donor organs. Pending success of alternative new preservation methods might justify further clinical evaluation of oxygen persufflation as safe, cheap, and easy applicable reconditioning method in liver allografts subgroups. 

## Figures and Tables

**Table 1:** Donor and recipient data; values given as median and range, where appropriate, in brackets.

Parameter	OPAL (*n* = 57)	Control (*n* = 59)	*p*-Value
Donor Age (years)	64 (30–95)	63 (28–84)	0.57
Donor Gender (m/f) (%)	56/44	44/56	0.27
Donor BMI (kg/m^2^)	25(19–42)	26 (19–51)	0.38
Donor ICU stay (days)	3 (1–16)	4 (1–19)	0.02
Donor Cause of death (n) Cerebrovascular Hypoxia Trauma Others	37 10 5 5	39 13 4 3	0.8
Donor aspartate aminotransferase (AST) (U/L)	48 (9–501)	41 (9–607)	0.8
Donor ALT (U/L)	32 (6–956)	33 (6–282)	0.87
Donor γGT (U/L)	46 (7–381)	57 (6–416)	0.29
Donor Sodium (µmol/L)	149 (132–163)	149 (132–169)	0.71
Donor Creatinin (µmol/L)	80 (32–689)	81 (33–265)	0.87
Donor Bilirubin (µmol/L)	9 (3.4–30)	8.2 (2.7–564)	0.16
Donor INR	1.13 (0.88–3.50)	1.12 (0.87–5.60)	0.81
Donor Risk Index	1.83 (1.1–2.5)	1.80 (1.1–2.5)	0.55
Allograft Histology (*n*) Macrosteatosis (≥20%) Microsteatosis (%)	49 13 50 (5–95)	47 6 40 (0–90)	0.09 0.36
Perfusion solution HTK/ UW (*n*)	53/4	52/7	0.37
Cold Ischemia Time (min)	443 (289–1090)	390 (259–740)	0.12
Recipient Age (years)	57 (31/69)	52 (24–67)	0.046
Recipient Gender (m/f) (%)	38/19	32/27	0.17
Recipient BMI (kg/m^2^)	27 (18–44)	25 (17–41)	0.14
Underlying disease (%) Viral Hepatitis HCC Cholestative disease Alcohol NASH Others	8 14 7 14 3 11	10 16 4 18 3 8	0.83
Charlson Co-morbidity Index	4 (1–8)	4 (2–8)	0.25
Laboratory Model for End-Stage Liver Disease (MELD)	13 (6–31)	15 (6–40)	0.16

**Table 2:** Secondary outcome parameters: values given as median and range, where appropriate, in brackets.

Parameter	OPAL (*n* = 57)	Control (*n* = 59)	*p*-Value
Retransplantation (*n*)	2	-	-
Early allograft dysfunction (EAD) (*n*)	14	12	0.58
Recipient ICU stay (days)	3 (1–45)	3 (1–41)	0.97
Post Tx dialysis (*n*)	5	9	0.28
Recipient hospital stay (days)	20 (2–114)	18 (1–85)	0.07
30-day mortality (*n*)	3	2	0.62
In-hospital mortality (*n*)	5	5	0.95
Postop. Comlications (*n*) Dindo-Clavien IIIa Dindo-Clavien IIIb Dindo-Clavien IVa Dindo-Clavien IVb	22 7 8 5 2	17 5 3 6 3	0.26
Rejection within 3 months (*n*)	6	8	0.61

**Table 3:** Early allograft dysfunction; censored for treatment arm: Nominal logistic analysis, multivariate analysis and likelihood ratio test (*p* values).

Parameter	OPAL		Control	
	Univariate	Multivariate	Univariate	Multivariate
Donor Age (years)	0.81		0.41	
Donor Age > 70years	0.7		0.65	
Donor BMI	0.09	3.26 0.07	0.67	0.14 0.71
Donor cause of death	0.31		0.23	
Donor ICU stay (days)	0.49		0.79	
Donor cardiopulmonary resuscitation	0.47	0.12 0.73	0.07	6.02 0.01
Donor AST (U/L)	0.87		0.1	
Donor ALT U/L)	0.38	2.54 0.11	0.03	5.25 0.02
Donor γGT (U/L)	0.73	0.97 0.32	0.03	1.25 0.26
Donor Bilirubin (µmol/L)	0.26		0.29	
Donor Risk Index	0.77		0.4	
Allograft histology: macrosteatosis	0.18		0.67	
Allograft histology: fibrosis	0.48		0.76	
Preservation solution	0.13	1.75 0.19	0.07	1.67 0.2
Cold Ischemia Time (h)	0.13		0.11	
Warm Ischemia Time (min)	0.61		0.66	
Duration of Surgical Procedure (min)	0.89		0.86	
Recipient Age (years)	0.36		0.6	
Recipient BMI	0.37	1.39 0.24	0.08	3.24 0.07
Lab-MELD score	0.52		0.43	

**Table 4:** Cox proportional hazard analysis (*p*-values), censored for long term patient survival and treatment arm.

Parameter	OPAL		Control	
	Univariate	Multivariate	Univariate	Multivariate
Donor age(years)	0.63	0.138 0.71	0.051	0.48 0.49
Donor age > 70 years	0.22		0.047	
Donor BMI	0.97		0.6	
Donor cause of death	0.17		0.99	
Donor ICU stay (days)	0.29		0.3	
Donor cardiopulmonary resuscitation	0.46		0.19	
Donor AST U/L)	0.87		0.53	
Donor ALT (U/L)	0.2		0.12	
Donor γGT (U/L)	0.67		0.69	
Donor Bilirubin µmol/L)	0.2		0.18	
Donor Risk Index	0.35		0.65	
Allograft histology: macrosteatosis	0.46	0.38 0.54	0.07	6.2 0.01
Allograft histology: fibrosis	0.09	1.17 0.28	0.44	3.69 0.06
Preservation solution	0.37		0.7	
Cold Ischemia Time (h)	0.55	0.05 0.83	0.09	3.55 0.06
Warm Ischemia Time (min)	0.83		0.27	
Duration of Surgical Procedure (min)	0.28		0.9	
Recipient Age (years)	0.71		0.17	
Recipient BMI	0.48		0.34	
MELD	0.49	0.48 0.49	0.054	5.06 0.03
